# Systemic Oxidative and Protein Profile Dysregulation in Tobacco-Associated Oral Malignancy and Precancerous Conditions

**DOI:** 10.7759/cureus.102190

**Published:** 2026-01-24

**Authors:** Monika V Padol, Anupkumar W Gore, Sharvari Tadas, Ankita V Gadekar, Saloni Wankhede, Shivani Ahirrao, Seema Gupta

**Affiliations:** 1 Department of Public Health Dentistry, Jawahar Medical Foundation's Annasaheb Chudaman Patil Memorial Dental College, Dhule, IND; 2 Department of Periodontics, Dr. Hedgewar Smruti Rugna Seva Mandal's Dental College and Hospital, Hingoli, IND; 3 Department of Orthodontics, Kothiwal Dental College and Research Centre, Moradabad, IND

**Keywords:** biomarkers, glutathione, oral cancer, precancerous, protein

## Abstract

Introduction

Tobacco-related oral carcinogenesis is a complex multistep process characterized by early biochemical and metabolic disturbances that occur before the appearance of clinically detectable diseases. This study aimed to assess and compare the systemic levels of glutathione (GSH), total plasma protein, and albumin among individuals with tobacco-associated oral cancer, individuals with potentially malignant oral disorders, and tobacco users without oral lesions.

Materials and methods

This cross-sectional study included 90 participants: 30 patients with oral cancer, 30 with potentially malignant oral disorders (oral submucous fibrosis and leukoplakia), and 30 healthy tobacco users serving as controls. Venous blood samples were collected under sterile conditions following a thorough clinical evaluation. Plasma GSH, total protein, and albumin concentrations were determined using standardized biochemical assays. Statistical analyses were then performed.

Results

Mean GSH levels were significantly elevated in the oral cancer group (9.23 ± 1.35 mg/dL) compared to the precancerous (4.78 ± 1.27 mg/dL) and control groups (2.67 ± 0.73 mg/dL) (p = 0.001). In contrast, total protein levels were highest among controls (7.25 ± 0.43 g/dL), followed by the precancerous group (6.78 ± 0.67 g/dL), and were lowest in patients with oral cancer (6.56 ± 0.47 g/dL) (p = 0.001). Albumin concentrations showed a similar declining trend, with significantly reduced values in the oral cancer group (3.43 ± 0.47 g/dL) compared to the precancerous (3.84 ± 0.47 g/dL) and control groups (4.00 ± 0.32 g/dL) (p = 0.001).

Conclusion

Alterations in systemic antioxidant status and protein profiles, particularly increased GSH and reduced albumin and total protein levels, may reflect early biochemical changes associated with malignant progression in tobacco users. Therefore, these markers may have potential utility in early risk assessment and preventive oral health interventions.

## Introduction

Oral health is a critical component of general health and well-being. Among the various diseases affecting the oral cavity, oral cancer is one of the most devastating due to its high morbidity and mortality, especially in countries such as India, where tobacco consumption is widespread. Oxidative stress is a central player in the progression from normal epithelium to premalignant lesion and eventually to malignancy. The reactive oxygen species (ROS) are produced due to the excessive stress induced by chewing tobacco, which damages DNA, proteins, and lipids [1,2]. This damage not only disrupts cellular homeostasis but also initiates mutagenesis and carcinogenesis in the affected cells. Biomarkers such as glutathione (GSH), total protein, and albumin are key indicators of oxidative stress and nutritional status in the body. GSH is an essential intracellular antioxidant, whereas albumin is a negative acute-phase protein and a significant indicator of inflammation and nutritional status [3-5]. Altered levels of these biomarkers have been implicated in several malignancies, including oral cancer. Monitoring these levels in individuals with tobacco habits could assist in the early detection of potentially malignant disorders and malignancies, thereby enabling timely intervention.

A previously published study by our group evaluated GSH, total protein, and albumin levels in smokeless tobacco users with oral precancerous and cancerous lesions. The study reported that GSH and albumin may serve as useful biomarkers for the early diagnosis of oral malignancies in this population [6]. Although the present study was conducted earlier, it was performed on an entirely independent cohort and included individuals with smoking, smokeless, and dual tobacco habits, thereby representing a broader and more comprehensive spectrum of tobacco exposure. No participants, samples, datasets, tables, or figures from previously published works were reused. Although similar biochemical parameters were assessed, the expanded exposure-based evaluation in the current study provides complementary evidence and enhances the clinical and public health relevance of the findings. Accordingly, this study aimed to evaluate and compare the levels of plasma GSH, total plasma protein, and plasma albumin in individuals with tobacco-induced oral cancer, individuals with precancerous conditions, and healthy controls.

## Materials and methods

Study design and setting

This study was conducted at the Department of Public Health Dentistry at Jawahar Medical Foundation's Annasaheb Chudaman Patil Memorial Dental College, Dhule. Data and sample collection were conducted prospectively over a defined period of 12 months (March 2019 to March 2020) to ensure consistency in procedural protocols and laboratory analyses.

Ethical approval and informed consent

The study protocol, along with the participant information sheet and informed consent documents, received approval from the Institutional Ethics Committee (IEC) of Jawahar Medical Foundation's Annasaheb Chudaman Patil Memorial Dental College before the commencement of the study (approval number: JMF's ACPMDC/IEC/PG/2019/753). Prior to enrolment, all participants were provided with a comprehensive explanation of the study objectives, procedures, potential benefits, and related aspects, following which written informed consent was obtained.

Study population and sample size estimation

The study comprised 90 participants, all adults aged between 18 and 65 years, with a verifiable history of tobacco use (smoking and/or smokeless forms). The participants were systematically categorized into three groups based on their clinical and histopathological diagnoses: Group A comprised 30 participants with histopathologically confirmed oral squamous cell carcinoma (OSCC); Group B comprised 30 participants diagnosed with clinically and histopathologically confirmed potentially malignant disorders, specifically oral submucosal fibrosis (OSMF) or leukoplakia; and Group C served as the control group and comprised 30 apparently healthy individuals with a history of tobacco use but no clinical evidence of any oral mucosal lesion or systemic illness.

The sample size was estimated using statistical power analysis software (G*Power 3.1, Heinrich-Heine-Universität Düsseldorf, Düsseldorf, Germany) based on data from prior pilot studies. With an anticipated effect size (f) of 0.4, alpha error of 0.05, and desired power of 80% for a one-way analysis of variance (ANOVA) comparing the three groups, the minimum required sample size was calculated as 90.

Inclusion and exclusion criteria

The inclusion criterion for all groups was a history of active tobacco use for a minimum of five years. Rigorous exclusion criteria were applied to minimize the confounding variables. Individuals were excluded if they had any known systemic illness that could alter oxidative stress or protein metabolism, such as diabetes mellitus, chronic renal or hepatic disorders, autoimmune diseases, or active infectious diseases. The participants with a history of chronic alcohol addiction, those who had undergone any form of cancer therapy (radiotherapy, chemotherapy, or surgery for malignancy) within the past five years, or those currently on antioxidant supplements were also excluded to ensure that the biochemical profiles reflected the conditions under investigation.

Sample collection and processing

Following a comprehensive clinical oral assessment conducted utilizing standard diagnostic tools (such as a mouth mirror, probe, and appropriate lighting), venous blood (5 mL) was procured from the antecubital vein of each participant under stringent aseptic conditions. The blood specimens were collected into vacutainers containing ethylenediaminetetraacetic acid (EDTA) and were promptly placed on ice prior to being transported to the central laboratory. The processing of the samples commenced within 30 minutes post-collection, during which the blood underwent centrifugation at 3000 revolutions per minute (rpm) for 15 minutes at 4°C to isolate plasma. The resulting plasma was meticulously transferred into sterile Eppendorf tubes to reduce the incidence of repeated freeze-thaw cycles and was subsequently preserved at -80°C until biochemical analysis, thereby ensuring the integrity of the analytes, particularly GSH [6].

Biochemical assays

All biochemical analyses were performed on stored plasma samples using standardized validated methods on a semiautomated biochemical analyzer. The quantification of GSH (mg/dL) was performed using a spectrophotometric assay, which is based on the detection of yellow 5-thio-2-nitrobenzoic acid (TNB) anion, produced by the interaction of GSH with 5,5'-dithiobis(2-nitrobenzoic acid) (DTNB) [7]. The total protein concentration (g/dL) was also assessed using a spectrophotometric assay, wherein peptide bonds in proteins interact with copper ions in an alkaline environment to produce a violet-hued chelate [8]. Albumin was measured (g/dL) through the bromocresol green (BCG) dye-binding technique at 630 nm, which demonstrates high specificity for albumin [9]. All reagents utilized were of analytical grade, and commercially available assay kits were employed following the manufacturer's guidelines. Each sample was analyzed in duplicate, and the average value was utilized for statistical evaluation. Concurrently, internal quality control sera with established values were analyzed within each batch to guarantee the accuracy and reliability of the assays.

Data collection and statistical analysis

Demographic, clinical, and biochemical data were systematically recorded using a predesigned form. Data management and statistical analyses were performed using a statistical software (IBM SPSS Statistics software, version 22.0, IBM Corp., Armonk, NY). Descriptive statistics were computed for all variables, and continuous data (such as GSH, albumin, and total protein levels) were expressed as the mean ± standard deviation (SD) for each study group. The normality of the data distribution was assessed using the Shapiro-Wilk test. One-way analysis of variance (ANOVA) was used as the primary test for intergroup comparisons of biochemical parameters across the three groups. In cases where ANOVA indicated a statistically significant difference (p < 0.05), a post hoc analysis was conducted using Tukey's honestly significant difference (HSD) test to identify the specific pairs of groups that were significantly different from each other. A p-value of less than 0.05 was considered statistically significant for all inferential tests conducted in the study.

## Results

The demographic analysis of the 90 participants showed comparable distributions of sex, age, and tobacco habits among the three groups. The number of men was 18 (60%) in Group 1 (OSCC), 21 (70%) in Group 2 (premalignant), and 15 (50%) in Group 3 (controls), with no statistically significant difference (p = 0.286). Similarly, the pattern of tobacco use, six maximum smokers (20%) in Group 3, 12 smokeless (40%) in Group 1, or 18 in both (60%) in Group 2, did not differ significantly between the groups (p = 0.649). However, a statistically significant difference in the mean age was observed (p = 0.056). Group 2 had the highest mean age (37.80 ± 5.12 years), followed by Group 3 (35.24 ± 6.45 years) and Group 1 (34.45 ± 4.52 years), respectively. Therefore, the groups were well-matched for sex, age, and the type of tobacco consumption. Group 1 had a maximum mean duration of tobacco use compared to the healthy controls (Table 1).

**Table 1:** Demographic details of each group. Values are presented as mean ± standard deviation (SD) for age and the duration of tobacco use and frequency (percentage) for sex and tobacco habit. Test value obtained with an independent t-test for continuous data and a chi-square test for categorical data. *P < 0.05 denotes statistical significance. OSCC: oral squamous cell carcinoma

Parameters	Category	Group 1 (OSCC)	Group 2 (premalignant)	Group 3 (control)	Test value	P-value
Sex	Male	18 (60%)	21 (70%)	15 (50%)	2.57	0.286
	Female	12 (40%)	9 (30%)	15 (50%)		
Age (mean ± SD)	Years	34.45 ± 4.52	37.80 ± 5.12	35.24 ± 6.45	3.12	0.056
Tobacco habit	Smoke	3 (10%)	3 (10%)	6 (20%)	2.40	0.649
	Smokeless	12 (40%)	9 (30%)	9 (30%)		
	Both	15 (50%)	18 (60%)	15 (50%)		
Duration of tobacco use (mean ± SD)	Years	14.50 ± 3.50	7.80 ± 5.60	6.24 ± 4.15	4.52	0.015*

One-way ANOVA revealed statistically significant differences in all biochemical parameters across the three groups (p = 0.001). The analysis indicated a significant progressive decline in plasma GSH levels (mg/dL) from Group 1 (9.23 ± 1.35) to Group 3 (2.67 ± 0.73), with the lowest levels observed in the healthy control group. Conversely, both total protein (g/dL) and albumin (g/dL) levels demonstrated a significant increasing trend from Group 1 to Group 3 (protein: 6.56-7.25; albumin: 3.43-4.00). The exceptionally high F-value for GSH underscores the magnitude of the difference in antioxidant status. These findings indicate a state of significant oxidative stress, marked by depleted GSH levels, in patients with OSCC and premalignant lesions compared with controls, while the concurrent rise in protein and albumin levels may reflect a reactive systemic metabolic response or an acute-phase profile associated with the disease process (Table 2).

**Table 2:** Intergroup comparison of outcome variables using a one-way analysis of variance (ANOVA) test. Values are presented as mean and standard deviation (SD). *P = 0.001 denotes statistically significant values. GSH, glutathione; OSCC, oral squamous cell carcinoma

Variables	Group 1 (OSCC) (mean ± SD)	Group 2 (premalignant) (mean ± SD)	Group 3 (control) (mean ± SD)	F statistic	P-value
GSH levels (mg/dL)	9.23 ± 1.35	4.78 ± 1.27	2.67 ± 0.73	300.624	0.001*
Total protein levels (g/dL)	6.56 ± 0.47	6.78 ± 0.67	7.25 ± 0.43	14.746	0.001*
Albumin levels (g/dL)	3.43 ± 0.34	3.84 ± 0.47	4.00 ± 0.32	21.815	0.001*

Post hoc Tukey's test revealed distinct patterns for each biochemical marker. For GSH, all pairwise comparisons were statistically significant (p = 0.001), showing a pronounced and progressive depletion from Group 1 (OSCC: 9.23) to Group 2 (premalignant: 4.78) and Group 3 (control: 2.67). This indicates a severe and graded decline in the antioxidant capacity as the disease progresses. In contrast, protein levels showed a significant increase only when comparing the control group to both patient groups, with no difference between the OSCC and premalignant states (p = 0.190). Albumin followed a similar pattern, being significantly lower in OSCC than in the premalignant (p = 0.001) and control groups (p = 0.001) but not differing between the premalignant and control groups (p = 0.263). These results suggest that oxidative stress, marked by GSH depletion, is an early and escalating event in oral carcinogenesis, whereas alterations in protein and albumin metabolism manifest more distinctly in established malignancies than in the premalignant phase (Table 3).

**Table 3:** Pairwise comparison of groups using post hoc analysis with Tukey's test. *P = 0.001 denotes statistically significant values. GSH, glutathione; OSCC, oral squamous cell carcinoma

Variables	Pairwise comparison of groups	Mean difference	P-value
GSH (mg/dL)	Group 1 (OSCC) versus Group 2 (premalignant)	4.45	0.001*
	Group 1 (OSCC) versus Group 3 (control)	6.56	0.001*
	Group 2 (premalignant) versus Group 3 (control)	2.11	0.001*
Total protein (g/dL)	Group 1 (OSCC) versus Group 2 (premalignant)	0.22	0.190
	Group 1 (OSCC) versus Group 3 (control)	0.69	0.001*
	Group 2 (premalignant) versus Group 3 (control)	0.46	0.003*
Albumin (g/dL)	Group 1 (OSCC) versus Group 2 (premalignant)	0.42	0.001*
	Group 1 (OSCC) versus Group 3 (control)	0.57	0.001*
	Group 2 (premalignant) versus Group 3 (control)	0.15	0.263

Pearson's correlation analysis revealed significant relationships between the biochemical parameters and demographic/clinical variables. A strong negative correlation was observed between plasma albumin levels and health status (r = -0.72), indicating that declining health (e.g., progression toward malignancy) is associated with a significant decrease in albumin concentration. Similarly, GSH levels showed a strong positive correlation with health status (r = 0.65), with poorer health status correlating with higher GSH levels, which may reflect a compensatory antioxidant response in diseased states. Furthermore, the duration of tobacco use demonstrated a strong positive correlation with GSH (r = 0.56) and a moderate negative correlation with albumin (r = -0.48), suggesting that long-term tobacco exposure is linked to increased oxidative stress and altered protein metabolism. Weak and nonsignificant correlations with age imply that these biochemical changes are more directly related to disease pathology and tobacco use than to the aging process itself (Table 4).

**Table 4:** Pearson's correlation (r) analysis between biochemical parameters and demographic/clinical variables. Data presented as correlation value (r) and significant p-value; r: 0.00-0.39, weak correlation; 0.40-0.79, moderate correlation; 0.80-1.00, strong correlation. Health condition: oral squamous cell carcinoma/premalignant disorder/healthy patient. *P < 0.05: significant. GSH: glutathione

Variables	Age (years)	Tobacco use (years)	Health condition
GSH	0.23 (p = 0.078)	0.56 (p = 0.021*)	0.65 (p = 0.003*)
Total protein	-0.35 (p = 0.081)	-0.38 (p = 0.042*)	-0.67 (p = 0.001*)
Albumin	-0.12 (p = 0.341)	-0.48 (p = 0.016*)	-0.72 (p = 0.022*)

## Discussion

This study aimed to compare the levels of GSH, total plasma protein, and albumin among individuals with tobacco-induced oral cancer, individuals with precancerous conditions, and healthy controls. The results demonstrated statistically significant alterations in biochemical markers across the three groups, highlighting their potential role in the early detection and monitoring of oral malignancies, especially in individuals with a history of tobacco consumption.

A striking observation was the elevated blood GSH levels in patients with cancer compared to those in the precancerous and control groups. This increase was statistically significant. Although GSH is conventionally regarded as a protective antioxidant, its overexpression in tumor cells has been linked to enhanced detoxification and tumor progression. This study aligns with the findings of Wong et al., who found elevated GSH levels in oral cancer tissues compared to adjacent normal tissues and healthy mucosa, suggesting that cancer cells may upregulate antioxidant defense mechanisms to support uncontrolled proliferation [4]. This paradoxical increase can be interpreted as a survival strategy employed by malignant cells under constant oxidative stress, often triggered by carcinogens such as tobacco [3-5,10]. Oxidative stress, primarily through the excessive production of ROS, plays a key role in cancer pathogenesis. Elevated GSH levels neutralize ROS, maintaining intracellular redox balance and aiding tumor cell survival. However, it is important to note that previous studies have reported contradictory findings, where GSH levels were lower in OSCC than in leukoplakia and controls [11]. These discrepancies may be attributed to differences in sample size, study design, or cancer progression stage. Nevertheless, the current findings reinforce the potential diagnostic value of elevated GSH levels in distinguishing oral cancer from precancerous and normal states [12-15].

The study also demonstrated a gradual decrease in serum albumin and total protein levels from controls to patients with precancerous lesions. These findings are significant because they reflect the body's systemic response to malignant processes. Albumin, a negative acute-phase reactant, decreases in response to systemic inflammation, which is commonly observed in patients with cancer. As described in previous studies, the production of inflammatory cytokines, such as interleukin-6 (IL-6) and tumor necrosis factor (TNF), can suppress hepatic albumin and enhance vascular leakage, leading to hypoalbuminemia [16-18]. Albumin is a major extracellular antioxidant. It scavenges free radicals via its free thiol group (Cys34), making it susceptible to oxidative stress. The decline in albumin levels observed in this study may also be due to oxidative modifications, as supported by Chandran et al., who showed a significant increase in oxidative protein products and a corresponding reduction in antioxidant status in patients with oral cancer [19].

The progressive alteration of these biomarkers across the three groups not only indicates their potential as diagnostic tools but also reflects the severity of the disease. The significant correlation between these biomarkers and tobacco use duration, as presented in the results, supports their application as early warning indicators in high-risk populations. For instance, GSH may serve as a marker of tumor burden and cellular adaptation to oxidative stress, whereas declining albumin levels may signal systemic inflammation and malnutrition, both of which are hallmarks of cancer progression. The utility of albumin and GSH as prognostic markers has been proposed in other studies, where their levels were significantly altered in patients with leukoplakia and OSCC [11].

Glutathione plays a central role in cellular defense against oxidative stress through the GSH-glutathione disulfide (GSSG) redox cycle. Under conditions of sustained ROS generation induced by tobacco exposure, GSH is oxidized to glutathione disulfide (GSSG) while detoxifying peroxides, thereby protecting cellular macromolecules from oxidative injury [20]. The observed elevation of plasma GSH levels in patients with oral cancer in the present study likely represents a compensatory systemic response to persistent oxidative stress rather than a reduction in oxidative burden. Similar findings have been reported in malignant conditions, where tumor cells upregulate GSH synthesis to maintain redox balance, support proliferation, and enhance resistance to oxidative damage and apoptosis [4,10,21].

Albumin and total plasma protein serve as important indicators of systemic oxidative stress and inflammatory status. Albumin acts as a major extracellular antioxidant via its free thiol group and is particularly vulnerable to oxidative modification during chronic inflammatory states [14,15]. The significant reduction in albumin and total protein levels observed in patients with oral cancer may therefore reflect oxidative protein damage and the cytokine-mediated suppression of hepatic protein synthesis rather than nutritional deficiency alone [16-19]. While tobacco exposure induces localized oxidative stress within the oral mucosa, the present study evaluated plasma biomarkers, which primarily reflect systemic oxidative stress. Thus, the altered protein and albumin profiles support the concept that tobacco-associated oral malignancy is accompanied by a generalized systemic redox imbalance in addition to local tissue injury [13,19].

The differing correlation patterns observed between GSH, total protein, and albumin can be explained by their distinct biological roles and regulation under oxidative stress. While GSH is a dynamic intracellular antioxidant that is actively upregulated in response to oxidative burden, total protein and albumin reflect broader systemic inflammatory and metabolic changes. Albumin, in particular, is a negative acute-phase protein and a major extracellular antioxidant that is preferentially depleted during chronic inflammation and oxidative stress. Consequently, the positive correlation of GSH with disease severity and tobacco exposure, alongside the negative correlation of albumin and total protein, reflects parallel but biologically divergent adaptive responses to systemic oxidative stress, rather than contradictory findings.

Clinical relevance and applications

From a clinical standpoint, the integration of biochemical markers, such as GSH, total protein, and albumin, into routine screening protocols can significantly improve early diagnosis, particularly in tobacco populations, where the risk of malignant transformation is high. While histopathology remains the gold standard, biochemical screening provides a noninvasive and cost-effective adjunct that can guide clinicians in identifying high-risk individuals for further evaluation [13,18]. Furthermore, monitoring these biomarkers can help assess treatment response and disease progression. This study describes the role of GSH in chemoresistance, where its upregulation may hinder the efficacy of certain therapies. Thus, strategies to modulate GSH levels should be explored as potential therapeutic interventions [19-23].

Limitations

Despite the meaningful observations generated by this investigation, several constraints should be considered when interpreting the results. The study population, while sufficient for exploratory analysis, may restrict the broader applicability of the findings, underscoring the need for future research with larger, multicenter cohorts. Furthermore, the cross-sectional design precluded the establishment of cause-effect relationships and prevented the evaluation of temporal variations. The incorporation of a wider panel of oxidative stress and inflammatory biomarkers, including C-reactive protein, malondialdehyde, and ceruloplasmin, would enhance the understanding of biochemical changes associated with carcinogenic processes. In addition, the longitudinal assessment of these markers before and after therapeutic interventions may contribute to identifying potential targets for personalized treatment approaches.

A further limitation of the present study is the reliance on plasma GSH levels as the primary indicator of oxidative stress. Although GSH is a well-established systemic antioxidant marker, its concentration may be influenced by compensatory upregulation in response to chronic oxidative stress, particularly in malignant states. Additionally, plasma GSH levels do not directly reflect localized tissue-level oxidative damage within the oral mucosa. The absence of complementary oxidative stress markers, such as lipid peroxidation products, oxidative protein modifications, or inflammatory biomarkers, limits the ability to comprehensively characterize oxidative stress severity and progression. Future studies incorporating a broader biomarker panel and tissue-specific assessments would allow more precise differentiation between localized and systemic oxidative stress.

## Conclusions

Biochemical markers such as GSH, total plasma protein, and albumin can be effectively utilized for the early detection of precancerous and cancerous changes in tobacco users. Monitoring these markers may help identify high-risk individuals and enable timely intervention strategies. The routine assessment of GSH, albumin, and total protein levels in tobacco users can serve as an early warning system for the onset of oral premalignant and malignant lesions. This could play a vital role in preventive public health strategies and improve long-term outcomes through early diagnosis.

## References

[1] Zhang L, Mock D (1991). Alteration in glutathione level during carcinogenesis of hamster buccal pouch mucosa. J Dent Res.

[2] Seo YS, Park JM, Kim JH, Lee MY (2023). Cigarette smoke-induced reactive oxygen species formation: a concise review. Antioxidants (Basel).

[3] Kolagal V, Karanam SA, Dharmavarapu PK (2009). Determination of oxidative stress markers and their importance in early diagnosis of uremia-related complications. Indian J Nephrol.

[4] Wong DY, Hsiao YL, Poon CK, Kwan PC, Chao SY, Chou ST, Yang CS (1994). Glutathione concentration in oral cancer tissues. Cancer Lett.

[5] Balasenthil S, Saroja M, Ramachandran CR, Nagini S (2000). Of humans and hamsters: comparative analysis of lipid peroxidation, glutathione, and glutathione-dependent enzymes during oral carcinogenesis. Br J Oral Maxillofac Surg.

[6] Nimbal A, Ahirrao B, Vishwakarma A, Vishwakarma P, Wani AB, Patil AA (2024). Comparative evaluation of GSH, total protein and albumin levels in patients using smokeless tobacco with oral precancerous and cancerous lesions. Med Int (Lond).

[7] Beutler E, Duron O, Kelly BM (1963). Improved method for the determination of blood glutathione. J Lab Clin Med.

[8] Wokes F, Still BM (1942). The estimation of protein by the biuret and Greenberg methods. Biochem J.

[9] Rodkey FL (1965). Direct spectrophotometric determination of albumin in human serum. Clin Chem.

[10] Saroja M, Balasenthil S, Nagini S (1999). Tissue lipid peroxidation and glutathione-dependent enzyme status in patients with oral squamous cell carcinoma. Cell Biochem Funct.

[11] Shetty SR, Babu S, Kumari S, Shetty P, Vijay R, Karikal A (2013). Serum glutathione levels in oral leukoplakia and oral squamous cell carcinoma- a clinicopathological study. Am J Can Prev.

[12] Manoharan S, Kolanjiappan K, Suresh K, Panjamurthy K (2005). Lipid peroxidation & antioxidants status in patients with oral squamous cell carcinoma. Indian J Med Res.

[13] Choudhari SK, Chaudhary M, Gadbail AR, Sharma A, Tekade S (2014). Oxidative and antioxidative mechanisms in oral cancer and precancer: a review. Oral Oncol.

[14] Oettl K, Stauber RE (2007). Physiological and pathological changes in the redox state of human serum albumin critically influence its binding properties. Br J Pharmacol.

[15] Gutteridge JM (1986). Antioxidant properties of the proteins caeruloplasmin, albumin and transferrin. A study of their activity in serum and synovial fluid from patients with rheumatoid arthritis. Biochim Biophys Acta Protein Struct Mol Enzymol.

[16] Simons JP, Schols AM, Buurman WA, Wouters EF (1999). Weight loss and low body cell mass in males with lung cancer: relationship with systemic inflammation, acute-phase response, resting energy expenditure, and catabolic and anabolic hormones. Clin Sci (Lond).

[17] O'Gorman P, McMillan DC, McArdle CS (1998). Impact of weight loss, appetite, and the inflammatory response on quality of life in gastrointestinal cancer patients. Nutr Cancer.

[18] Barber MD, Ross JA, Fearon KC (1999). Changes in nutritional, functional, and inflammatory markers in advanced pancreatic cancer. Nutr Cancer.

[19] Chandran V, Anitha M, Avinash S, Rao GM, Shetty BV, Sudha K (2012). Protein oxidation: a potential cause of hypoalbuminemia in oral cancer. Biomed Res.

[20] Meister A, Anderson ME (1983). Glutathione. Annu Rev Biochem.

[21] Richie JP Jr, Kleinman W, Marina P, Abraham P, Wynder EL, Muscat JE (2008). Blood iron, glutathione, and micronutrient levels and the risk of oral cancer. Nutr Cancer.

[22] Hiemstra PS (2002). The adaptive response of smokers to oxidative stress: moving from culture to tissue. Am J Respir Crit Care Med.

[23] Poli G, Leonarduzzi G, Biasi F, Chiarpotto E (2004). Oxidative stress and cell signalling. Curr Med Chem.

